# Effect of Tendon Strip (FCR vs APL) on Outcome of CMC Thumb Joint Arthroplasty With Pyrocarbon Disk Interposition

**DOI:** 10.1177/15589447211040879

**Published:** 2022-01-27

**Authors:** Cecile Maria Cornelia Agnes van Laarhoven, Marcus Chen Yee Tong, Mark van Heijl, Arnold Herman Schuurman, Brigitte Egeberta Petronella Adriana van der Heijden

**Affiliations:** 1 Erasmus Medical Center, Rotterdam, The Netherlands; 2 University Medical Center Utrecht, The Netherlands; 3 Jeroen Bosch Hospital, ‘s-Hertogenbosch, The Netherlands; 4 Diakonessenhuis, Utrecht, The Netherlands; 5 Radboud University Medical Center, Nijmegen, The Netherlands

**Keywords:** PyroDisk, interposition arthroplasty, carpometacarpal thumb joint, osteoarthritis, trapeziometacarpal arthritis, tendon strip, suspensionplasty

## Abstract

**Background:**

Pyrocarbon disk interposition for carpometacarpal (CMC) thumb joint osteoarthritis can be performed with a flexor carpi radialis (FCR) or abductor pollicis longus (APL) tendon strip. With the FCR technique, a ligament reconstruction is performed in addition to disk fixation, whereas with the APL technique the disk is simply secured in place. Our aim is to compare long-term postoperative outcomes between both techniques.

**Methods:**

In this observational study, we included 106 patients in 2 centers operated on between 2006 and 2011. We assigned patients to the FCR group or the APL group based on the respective tendon strip used. As a primary outcome, we analyzed postoperative key pinch. In addition, we analyzed postoperative tip pinch and tripod pinch, grip strength, range of motion, thumb height maintenance, and patient-reported outcome measures (PROMs).

**Results:**

The analysis showed clinically important stronger key pinch for the APL group (β = 1.28 kg). Tip pinch and grip strength showed higher outcome for the FCR group (β = 1.22 kg and 5.14 kg, respectively). Palmar abduction was in favor of the FCR group and opposition in favor of the APL group, but these were interpreted as not clinically relevant. Radiological thumb height maintenance and PROMs showed no clinical difference.

**Conclusions:**

Pyrocarbon disk interposition arthroplasty for CMC thumb joint osteoarthritis can be secured with an APL or FCR tendon strip. At long-term follow-up, use of an APL tendon strip results in significantly higher key pinch and better opposition. Tip pinch, grip strength, and palmar abduction were better after use of the FCR tendon strip. The choice of the tendon strip can be based on outcomes considered most important for the individual patient.

## Introduction

Most techniques described for carpometacarpal (CMC) thumb joint osteoarthritis strive to achieve pain reduction with preservation of as much range of motion (ROM) and strength as possible.^1,2^ To date, trapeziectomy combined with a tendonplasty is still widely used, despite a higher complication rate than simple trapeziectomy with comparable clinical outcome.^3-7^ Many hand surgeons prefer to add a tendonplasty to a trapeziectomy to reduce the possibility of subsidence of the first metacarpal with abutment on the distal pole of the scaphoid, which may cause recurrent pain that is difficult to treat. Furthermore, subsidence is hypothesized to lead to less stability and loss of strength.^8^

When osteoarthritis solely affects the first CMC joint, the scaphotrapeziotrapezoidal (STT) joint can be spared by performing hemitrapeziectomy instead of a total trapeziectomy, thus contributing to the maintenance of thumb length and stability.^9,10^ A hemitrapeziectomy is also often combined with an interposition, most usually with autologous tendon, with various outcomes in terms of strength and maintenance of thumb length.^11^

In 2005, the PyroDisk (Integra LifeSciences Corporation, Plainsboro, New Jersey) was introduced as an interposition arthroplasty after distal hemitrapeziectomy.^12-14^ The interposition of the disk and the hardness of the pyrolytic carbon contribute to preservation of thumb length. The disk is secured in the joint space with a tendon strip, which contributes to alignment of the thumb with preservation of function and strength. Either a strip of flexor carpi radialis (FCR) tendon or abductor pollicis longus (APL) tendon can be used to secure the disk between the distal hemitrapezium and the first metacarpal. With the FCR, a ligament reconstruction comparable to that of the Burton-Pellegrini concept is performed in addition to fixation of the disk.^15^ With the APL, the disk is only fixed in its position without any ligament reconstruction or suspension of the thumb. Previous outcome studies after pyrocarbon disk interposition have shown good results in pain reduction and survival rate of the disk.^16^ A recent study, comparing the pyrocarbon disk interposition with trapeziectomy combined with ligament reconstruction, reported better key pinch after pyrocarbon disk interposition.^14^ The effect of the tendon used for fixation of the pyrocarbon disk on clinical outcomes has not been studied before.

To analyze the differences in the use of an FCR or APL tendon strip for pyrocarbon disk interposition, we conducted a comparative study. We analyzed key pinch as our primary outcome and tested whether the use of an FCR tendon strip results in higher key pinch than the use of the APL tendon strip. Secondarily, we analyzed differences in tip pinch and tripod pinch, power of grip, ROM, thumb height maintenance, and patient-reported outcome measures (PROMs).

## Patients and Methods

### Study Design and Patients

This observational cohort study is reported following the Strengthening the Reporting of Observational Studies in Epidemiology statement.^17^ It describes retrospectively gathered data and is part of a multicenter study by van Laarhoven et al^18^ on outcomes after pyrocarbon disk interposition arthroplasty for the treatment of CMC thumb joint osteoarthritis. Previously, we focused on PROMs, complications, and survival rate of pyrocarbon disk interposition arthroplasty after medium-term to long-term term follow-up.^18^ In this study, we focus on the differences between the 2 securing techniques for pyrocarbon disk interposition arthroplasty. We obtained assessments of patients operated on between 2006 and 2011, after approval from the institutional review board and written informed consent from all study participants. We assigned patients to the FCR group or the APL group based on the tendon strip used for securing the pyrocarbon disk. Surgeon preference determined which tendon strip was used. Inclusion criteria for this study were CMC thumb joint osteoarthritis stage 2 or 3 Eaton and Glickel^19^ and nonresponsiveness to nonoperative treatment for at least 3 months at initial presentation. Exclusion criteria were techniques used other than the originally described pyrocarbon disk interposition with hemitrapeziectomy, hyperlaxity syndromes, systematic inflammatory arthritis (such as rheumatoid arthritis, gout, or psoriasis), and concomitant major surgery on the same hand. Due to scarcity of preoperative hand measurements, we used cross-sectional data gathered at 1 time point for both groups (during long-term follow-up). We collected demographic data and perioperative complications by review of medical records.

### Surgical Technique

**Figure 1.**
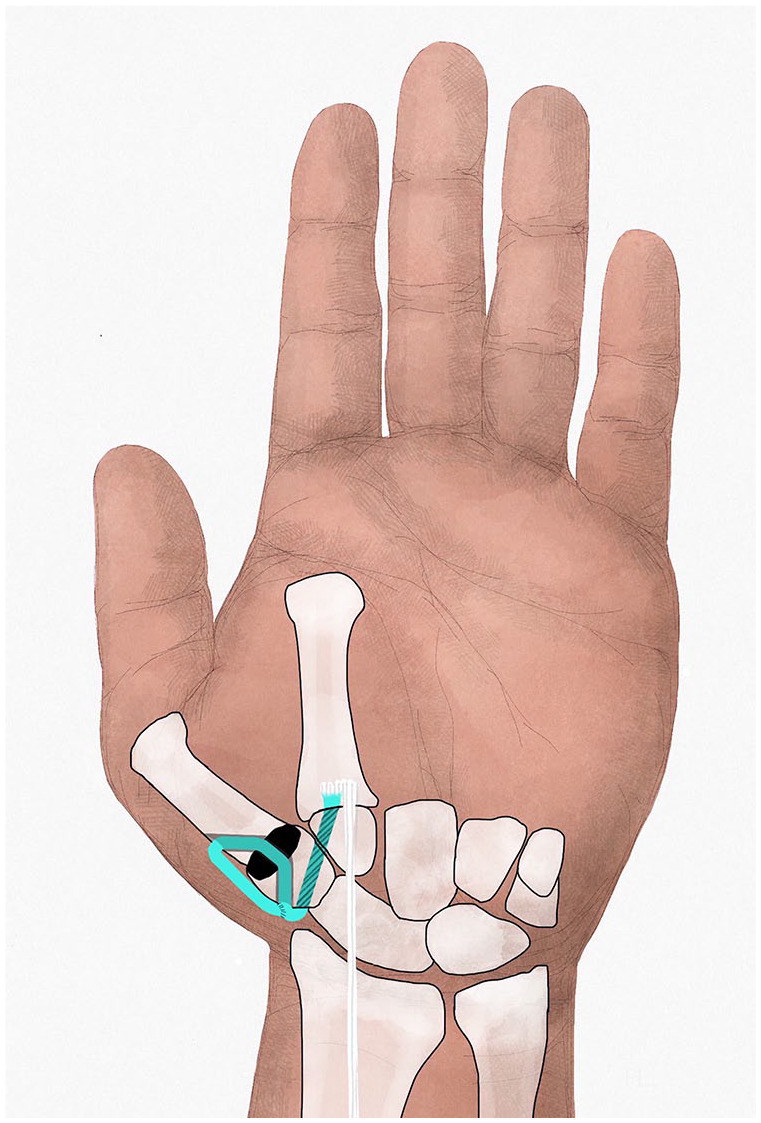
Flexor carpi radialis tendon strip used for securing pyrocarbon disk.

**Figure 2.**
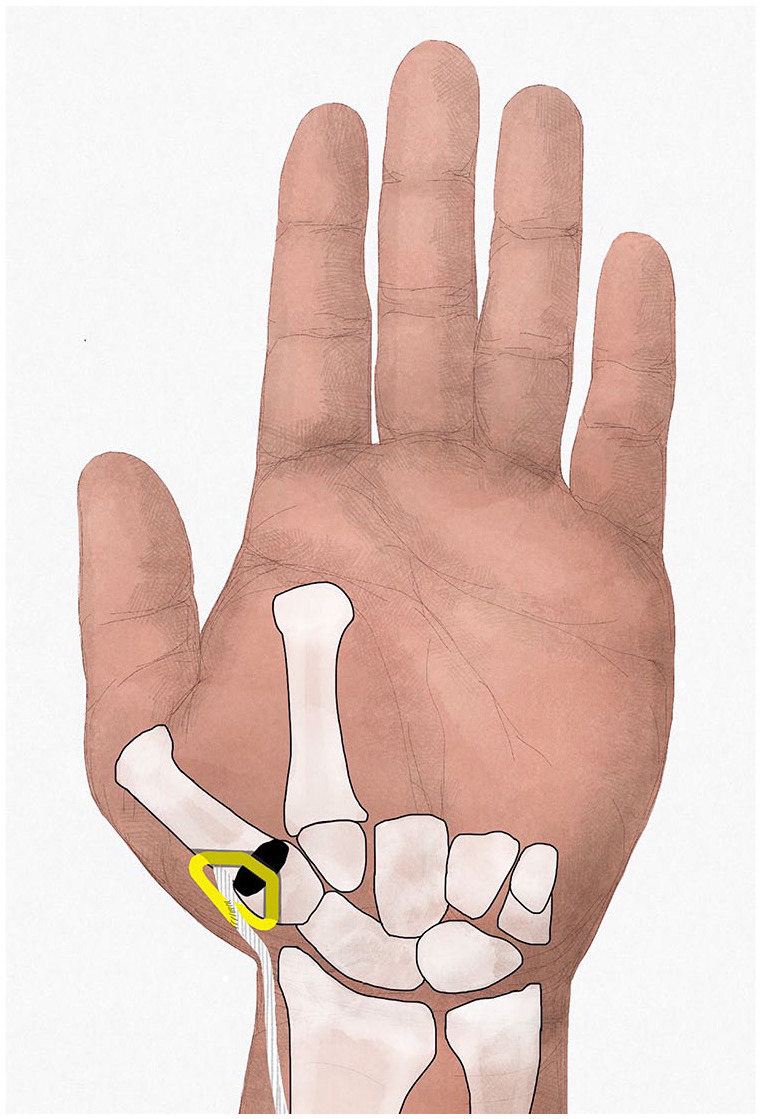
Abductor pollicis longus tendon strip used for securing pyrocarbon disk.

Pyrocarbon disk interposition arthroplasty was performed as previously described.^18^ By a middorsal longitudinal incision along the first metacarpal and trapezium, the CMC thumb joint is approached. The base of the first metacarpal and the distal half of the trapezium are resected, transverse to the longitudinal axis of the first metacarpal and parallel to each other. Subsequently, bony tunnels are created in the hemitrapezium (from dorsal-proximal to central-distal) and the base of the first metacarpal (from central-proximal to dorsal-distal), equal for both techniques. After determining the proper implant diameter by the diameter of the first metacarpal base, either the FCR or the APL tendon strip is harvested to secure the disk in its position in the intertrapezial space (Figures 1 and 2).

#### FCR technique

The FCR tendon strip is harvested by 2 or 3 incisions over the FCR tendon on the volar side of the forearm (Figure 1). The tendon strip (one-third to half of tendon width and 5-10 cm of tendon length) is cut proximally and released up to its insertion distally, leaving the insertion intact. Passing from volar to dorsal, deep to the first dorsal compartment tendons, the tendon strip is passed through the tunnel in the hemitrapezium, the disk, and the tunnel in the proximal metacarpal. The tendon strip is tightened to secure the disk, and the residual tendon is then folded back and sutured to itself and to the periosteum of the metacarpal base, before being subsequently incorporated with the capsular closure using absorbable sutures for additional fixation of the disk. Because of its length, the FCR strip can be folded back and forth many times to reenforce the capsule if necessary. As the insertion of the FCR tendon strip is left intact on the volar second metacarpal base, a volar to dorsal ligament reconstruction and suspension of the first on the second metacarpal base is performed in addition to this fastening technique.

#### APL technique

The APL tendon strip is harvested under direct vision via the same incision as used for resection of the CMC thumb joint (Figure 2). A strip of APL tendon or one of the extra APL tendons is harvested at maximum length. After release of the first extensor compartment, the APL strip is cut proximally and released to its distal insertion, leaving the insertion intact. The strip is passed through the tunnels in the hemitrapezium, the disk, and the first metacarpal base and sutured to itself and the periosteum of the first metacarpal base under gradual tension with absorbable sutures. Because the insertion of the APL is on the radial side of the metacarpal base, a dorsal ligament reconstruction is performed by securing the disk with the APL tendon strip.

For both procedures, the position of the disk is assessed with radiography during the procedure. A thumb spica cast is applied for 4 weeks of immobilization. Hand therapy is focused on unloaded ROM during the first 4 weeks after operation, followed by loaded ROM exercises thereafter. Immobilization and hand therapy protocol were similar for both groups.

### Measurements

**Figure 3.**
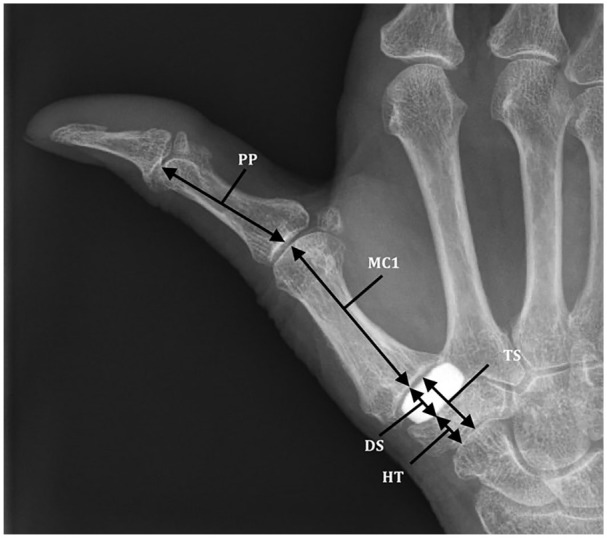
Thumb height measurements. *Note.* The ratio used is (HT + DS + MC1/PP). Because the disk articulates with the HT and MC1, both can be accountable for height loss of the thumb and are taken in account on the measurement. We calculated a ratio of thumb length to correct for differences in hand size and radiology. A loss in ratio of 20% is accounted as clinically relevant. HT = hemitrapezium, measured from the distal joint surface of the scaphoid to the proximal surface of the implant; DS = disk space, measured from the distal joint surface of the HT to the joint surface first metacarpal base; TS = trapezial space, measured between the distal joint surface of the scaphoid and the joint surface of the base of the first metacarpal; MC1 = first metacarpal; PP = proximal phalanx, measured from midpoint of the proximal and distal joint surfaces.

For the primary outcome, we measured key pinch strength using baseline pinch gauge (E-link H500 Hand Kit; Biometrics Ltd, Gwent, UK). Secondarily, we analyzed tip pinch and tripod pinch by baseline pinch gauge and grip strength (Jamar) using a hydraulic hand dynamometer in position 2 (H500 Hand Kit; Biometrics Ltd). The ROM was analyzed with Pollexograph for palmar abduction and Kapandji for thumb opposition.^20,21^ The average of 3 subsequent measurements was used for the analysis.^22,23^ Two trained research assistants performed all strength and ROM measurements. In addition, we measured thumb height on radiographs taken directly postoperatively and at long-term follow-up. We measured thumb height as the ratio of the thumb length (sum of hemitrapezium, disk height, and metacarpal height) and the proximal phalanx, measured on a lateral radiograph, as described by van Laarhoven et al^18^ (Figure 3). We used the difference between the ratios of direct postoperative and long-term follow-up as variable.

To measure pain, function, and satisfaction, we used Patient-Rated Wrist/Hand Evaluation (PRWHE),^24^ Michigan Hand Outcomes Questionnaire (MHQ),^25^ Disabilities of the Arm, Shoulder, and Hand (DASH),^26^ and a satisfaction questionnaire.^18,27,28^ The MHQ and satisfaction questionnaire show difference in the left and right hand; the PRWHE and DASH do not make this differentiation. Therefore, we asked bilaterally operated patients to fill in the PRWHE for both hands separately. The bilaterally operated patients filled in the DASH only once because of the more generic nature of this questionnaire. In this patient cohort, no upper extremity pathology was present.

### Power Analysis

We performed a post hoc power analysis for our linear multivariate regression model based on our primary outcome key pinch. For a multivariable linear regression model with 6 predictors, a sample size of 96 (the total disks in situ of both groups), and a probability level set at .05, we calculated a power of 1.0 with an observed *R*^2^ of 0.582.

### Statistical Analysis

The Kolmogorov-Smirnov test and histograms were used to assess distribution of the variables. We reported descriptive statistics in values as means and range for normal distributed data, as median and interquartile range (IQR) for nonnormal distributed data, or as absolute values and proportions (%). The 2 groups were analyzed against demographics to identify differences between the groups with a χ^2^ test for binominal variables and with an independent *t* test (normal distributed) or Mann-Whitney *U* test (nonnormal distributed) for continuous variables. Furthermore, we evaluated outcomes of strength and ROM, together with difference in thumb length (ratio) with a multivariable linear regression analysis, adjusting for age, sex, bilateral complaints, dominant hand operated, and follow-up in years. These variables are chosen because they can affect the outcome measurements independent of the surgical treatment. We analyzed PROMs with a Mann-Whitney *U* test because of the nonnormal distribution of outcomes and residuals. A *P* value smaller than .05 was considered statistically significant.

## Results

For this study, 76 patients treated with the APL technique and 30 patients with the FCR technique could be included out of the previous cohort based on the above-mentioned criteria. Table 1 shows the demographic outcomes. The groups were comparably similar in demographics except for follow-up duration (FCR 9.4 vs APL 8.3 years, *P* < .001). The mean follow-up in years of the whole group was 8.3 (IQR, 7.4-9.4).

**Table 1.** Demographics.

Demographics	FCR group n = 30	APL group n = 76	*P* value
Male	12 (40%)	20 (26%)	.167
Female	18 (60%)	56 (74%)	
Bilateral complaints	10 (33%)	27 (36%)	.831
Dominant side operated	11 (37%)	27 (36%)	.912
Follow-up, median (IQR), y	9.4 (8.0-9.9)	8.0 (7.3-8.8)	.001
Age at operation, median (IQR), y	58.7 (53.1-67.2)	60.3 (54.3-68.1)	.897
Disk removed	1 (3%)	9 (12%)	.177
Tendon rupture/luxation	—	1 (1%)	
STT arthritis	1 (3%)	6 (8%)	
Persisting pain, no cause	—	2 (3%)	
Complaints before operation			
<1 y	1 (3%)	8 (11%)	
1-2 y	5 (19%)	16 (21%)	
2-5 y	12 (40%)	38 (50%)	
>5 y	9 (30%)	12 (16%)	
Missing	3 (10%)	2 (3%)	

*Note.* Independent *t* test for continuous normal distributed data, Mann-Whitney *U* test for continuous nonnormal distributed data, and χ^2^ test for binominal (non)normal distributed data. FCR = flexor carpi radialis; APL = abductor pollicis longus; IQR = interquartile range; STT = scaphotrapeziotrapezoidal.

In the APL group, 9 disks were removed due to clinical complaints—in 6 patients in the first 2 years (1 tendon rupture with disk dislocation, 2 for persisting pain without a cause, and 3 for STT arthritis) and in 3 patients after 2 years (attributed to STT arthritis). Revision surgery in the APL group consisted of trapeziectomy and ligament reconstruction with an FCR tendon strip. In the FCR group, 1 revision was needed due to STT arthritis (within 2 years after the initial surgery), which resulted in a trapeziectomy and suspensionplasty with a toe extensor.

For the comparative analysis, we used data available of patients with the disk still in situ (67 patients in the APL group and 29 patients in the FCR group). Table 2 shows unadjusted measurements for strength, thumb height, and ROM. In Table 3, the betas for the techniques were depicted, with the FCR group as baseline after multivariable linear regression. For our primary outcome, the APL group scored 1.28 kg higher key pinch compared with the FCR group (*P* = .003), adjusted for the variables in our model (Table 3).

**Table 2.** Unadjusted Outcome of Measurements.

Measurements	APL (n = 62^a^)	FCR (n = 26^a^)
Key pinch, kg^b^	5.0 (8.5)	4.1 (6.5)
Tip pinch, kg^b^	3.3 (8.5)	4.9 (7.1)
Tripod pinch, kg^b^	4.4 (5.2)	4.5 (8.3)
Jamar grip, kg^b^	24.0 (51.4)	31.4 (47.0)
Palmar abduction, deg^b^	43.8 (46)	55.6 (50)
Kapandji^c^	9.5 (9-10)	9.0 (8-10)
Difference in thumb height ratio^b^	0.044 (0.48)	0.145 (0.52)

*Note.* APL = abductor pollicis longus; FCR = flexor carpi radialis; IQR = interquartile range.

a Hand measurements were available for 62 of the 67 patients in the APL group and 26 of the 29 patients in the FCR group.

b Values are given in mean and range for normal distributed variables.

c Values are given in median and IQR for non-normal distributed variables.

**Table 3.** Multivariate Linear Regression Output for FCR Group in Different Hand Measurements.

Measurements	β (beta)	Confidence interval		Significance (P-value)
		Lower bound	Upper bound	
Key pinch	−1.276	−2.107	−0.445	.003*
Tip pinch	1.225	0.616	1.834	<.001*
Tripod pinch	−0.067	−0.826	0.691	.860
Jamar grip	5.136	0.318	9.953	.037*
Palmar abduction	11.463	6.557	16.368	<.001*
Kapandji	−1.341	−2.324	−0.357	.008*
Difference in thumb height ratio	0.117	0.051	0.184	.001*

*Note.* Linear regression models with beta given for treatment with FCR outcome as baseline. Models corrected for treatment (FCR or APL tendon strip), age, sex, bilateral complaints, dominant hand operated, and follow-up in years. Full models are available in the Supplemental Material. FCR = flexor carpi radialis; APL = abductor pollicis longus; Significant when P-value smaller than 0.05, indicated with *.

Tip pinch was 1.23 kg stronger for the FCR group (*P* < .001). For tripod pinch, the beta was small (0.067) and not significant. The FCR group showed more power of grip and wider palmar abduction. The APL group showed better opposition measured by Kapandji. The difference in thumb height ratio was higher for the FCR group than for the APL group (*P* = .001). This means better thumb height maintenance for the APL group at long-term follow-up, although with a small difference. For all multivariable linear regression models, follow-up duration showed no significant influence on outcome for pinch strength, grip strength, or ROM.

Table 4 shows that the outcomes of the different PROMs between both groups were comparable.

**Table 4.** Comparison of PROMs Based on FCR and APL.

PROM Questionnaires	APL (n = 66^a^)	FCR (n = 28^a^)	Significance (P-value)
DASH	18.3 (10-40.4)	9.2 (3.3-42.1)	.179
PRWHE			
Total	16.5 (2-40.3)	12.0 (0-34.9)	.329
Pain	10.0 (0-19.5)	6.3 (0-13.1)	.770
Function	6.5 (1.5-21.5)	9.5 (0-21.8)	.221
MHQ			
Function operated hand	70	75	.077
ADL	83.8	88.6	.229
Work	85.0	82.5	.734
Pain	80.0	85.0	.102
Aesthetics	100.0	96.9	0.293
Satisfaction	75.0	87.5	.377
MHQ total	76.0 (64.8-92.6)	81.6 (71.6-91.9)	.360
Satisfaction			
Satisfaction with result	9.0 (8-10)	9.0 (8-10)	.834
Main reason for operation solved	9.0 (8-10)	10.0 (8-10)	.243

*Note.* Values are given as median (IQR). Because of nonnormal distribution outcomes and residuals, comparison was made with Mann-Whitney *U* test. PROMs = patient-reported outcome measures; FCR = flexor carpi radialis; APL = abductor pollicis longus; DASH = Disabilities of the Arm, Shoulder, and Hand; PRWHE = Patient-Rated Wrist/Hand Evaluation; MHQ = Michigan Hand Outcomes Questionnaire; ADL = activities of daily living; IQR = interquartile range.

a PROMs were available for 66 of the 67 patients in the APL group and for 28 of the 29 patients in the FCR group.

Radiography analysis was performed on the radiographs taken at long-term follow-up (available in 63 patients for the APL group and in 29 patients for the FCR group). For the APL group, sclerosis and osteophytes around the disk were seen in 5 patients (8%), a subluxation of less than one-fourth of the disk in 9 patients (14%), and STT arthritis in 5 patients (8%), without significant clinical complaints in all. In the FCR group, 4 patients (14%) showed sclerosis and osteophytes around the disk on radiography and 3 patients (10%) showed mild subluxation (less than one-fourth of the disk), again without clinical complaints.

## Discussion

The purpose of this study was to compare the clinical and radiological outcome between 2 fixation techniques (FCR vs APL) used for pyrocarbon disk interposition for the treatment of CMC thumb joint osteoarthritis. For our primary outcome, a higher key pinch was found for the APL technique than for the FCR technique. The FCR technique showed better tip pinch and power of grip. Range of motion, thumb height maintenance, PROMs, and revision rate showed comparable outcomes between both tendon groups.

The use of the FCR tendon strip after trapeziectomy is numerously described in the literature, and most studies report favorable results.^29-33^ The FCR tendon strip for CMC thumb joint arthroplasties is popular because it is easy to harvest and is often broad, which makes splitting possible for use as a tendon graft. The line of pull from the insertion on the volar side of the second metacarpal to the basal thumb joint can be used to correct for dorso-radial translation of the first metacarpal base and to suspend the first metacarpal to the second metacarpal, aiming to prevent subsidence of the first metacarpal as much as possible. The need of an extra incision on the volar side of the wrist for harvesting the FCR can be considered a disadvantage. Furthermore, the use of the FCR can potentially harm radial wrist stability when the tendon strip is taken too broad and tendinitis in the residual tendon can occur.^34^ Another disadvantage is that if revision surgery is needed, stabilization or suspension is less feasible with the FCR, unless the FCR is made really thin or fully sacrificed, thereby risking harm to the radial stability of the wrist.^34^

The use of an APL tendon strip after trapeziectomy is primarily described by Thompson^35^ as a suspension technique. The use of an APL tendon for pyrocarbon interposition arthroplasty has the advantage of preserving the FCR tendon and its stabilizing function. In addition, the APL can be harvested via the same incision and usually consist of multiple tendon strips, wherefore using one of these strips usually does not usually hinder function. Nevertheless, harvesting the APL requires precaution not to damage the radial superficial nerve and the radial artery in the anatomical snuffbox region. If revision surgery is needed, no bridges are burnt, and the FCR is still in situ to be used for any kind of arthroplasty.

We chose key pinch as the primary outcome because it has been shown to be affected first in early CMC thumb joint osteoarthritis.^36^ This study showed that the APL group has a stronger key pinch than the FCR group (1.28 kg). This magnitude of difference was shown to be of clinical importance. A previous report showed that minimal clinical important differences for pinch strength in patients with CMC thumb joint osteoarthritis are around 0.35 kg for the right dominant hand.^37^ In contrast, a clinical important difference in tip pinch was found in favor of the FCR group (1.26 kg). Tripod pinch did not show any clinical relevance (0.06 kg).

The ROM was different between groups, with a beta in favor for the FCR group on palmar abduction and a beta in favor for the APL group on opposition. Although significant, the differences in both ROM measurements were small and not clinically important. Patient-reported outcome measurements did not show any differences between the groups. Although strength can be related to function, the measured differences were not reflected in the function measured with PROMs.

Preservation of thumb height is one of the main advantages of pyrocarbon disk interposition arthroplasty. The hardness of pyrolytic carbon prevents wear of the disk without compressing the bone and contributes to thumb height after interposition.^38^ As expected, our results show that thumb height is maintained for both techniques. The difference (although significant with 0.044 for APL and 0.143 for FCR) can be interpreted as clinically small and irrelevant. With a mean proximal phalanx height of 31 mm and a thumb height ratio difference of 0.143 and 0.044, the thumb height loss can be calculated as 4.7 mm and 1.4 mm for the FCR group and APL group respectively. We may conclude that both techniques have maintained thumb height. Furthermore, the comparable thumb height between the techniques shows that the additional suspension with the FCR technique is not beneficial for thumb height in this technique, and the maintained thumb height is related to the use of the pyrocarbon disk. Previous reports on thumb height questioned the importance of thumb height maintenance on clinical outcomes.^39,40^ These reports compared trapeziectomy and trapeziectomy with ligament reconstruction and tendon interposition, both with loss of thumb height. They concluded that the extent of thumb loss did not influence the outcome, but no comparisons were made with techniques objectively maintaining thumb height, such as the pyrocarbon disk interposition.

A recent study published in 2020 by Smeraglia et al described 40 patients after pyrocarbon disk interposition (without making distinction between APL and FCR techniques) with a minimum of 8-year follow-up. The reported results are comparable with the overall results of our study.^16^ Their survival rate was 94%, and all revisions were done in the first 2 years after the initial operation. The overall survival in our study is slightly lower (overall 91%). No differences in survival rates were found between the FCR group (97%) and the APL group (88%). In our report, more than 80% of the revision surgeries were performed in the first 2 years after placement of the disk. The revisions within 2 years were due to STT arthritis (1 patient in FCR group and 3 in the APL group), persisting pain without a cause (2 in the APL group), and 1 tendon rupture after a fall on the hand (1 in the APL group). All revisions after more than 2 years were due to STT arthritis. We observed no luxations leading to revision.

The limitations of our study are the cross-sectional nature of the study and lack of preoperative measurements. Furthermore, the APL group was double the size of the FCR group. To address these limitations, we used a linear regression and corrected for variables that may have an influence on the subjective and functional outcomes. The consequential post hoc power analysis for the multivariable regression showed a high power. Furthermore, the long-term follow-up of more than 8 years and the large sample add to the strength of our analysis.

The use of the APL tendon strip or FCR tendon strip in this study was based on surgeon’s preference. Besides the main goal of securing the disk, the FCR provides a ligament reconstruction and suspension (plus, if needed, a capsule augmentation). This potentially provides more stability than just securing the disk, as done with the APL technique. However, clinically both techniques have shown comparably good results on hand measurements, with a favor for the APL technique for key pinch and opposition and for the FCR technique for tip pinch, power of grip, and palmar abduction. Thumb height was better maintained (although with a clinical small difference) for the APL group, which questions the importance of suspension with the FCR technique on thumb height.

In conclusion, this report shows that the APL technique results in a clinically important stronger key pinch. In contrast, the FCR technique results in a clinically stronger tip pinch and power of grip. The other outcomes showed no clinically relevant differences between both techniques (thumb height maintenance, ROM, PROMs, and complications). The choice of tendon for pyrocarbon disk interposition can be made by shared decision, based on which donor site morbidity is acceptable and which outcome is most important for the patient: strength of key pinch or tip pinch and power grip.

## Supplemental Material

sj-pdf-1-han-10.1177_15589447211040879 – Supplemental material for Effect of Tendon Strip (FCR vs APL) on Outcome of CMC Thumb Joint Arthroplasty With Pyrocarbon Disk Interposition Click here for additional data file. Supplemental material, sj-pdf-1-han-10.1177_15589447211040879 for Effect of Tendon Strip (FCR vs APL) on Outcome of CMC Thumb Joint Arthroplasty With Pyrocarbon Disk Interposition by Cecile Maria Cornelia Agnes van Laarhoven, Marcus Chen Yee Tong, Mark van Heijl, Arnold Herman Schuurman and Brigitte Egeberta Petronella Adriana van der Heijden in HAND
