# Supplementary material for: Effect of Tendon Strip (FCR vs APL) on Outcome of CMC Thumb Joint Arthroplasty With Pyrocarbon Disk Interposition
Source: Hand (N Y). 2022 Jan 27;18(2 Suppl):87S–95S. doi: 10.1177/15589447211040879 (PMC10052627; doi:10.1177/15589447211040879)
Supplement: sj-pdf-1-han-10.1177_15589447211040879 – Supplemental material for Effect of Tendon Strip (FCR vs APL) on Outcome of CMC Thumb Joint Arthroplasty With Pyrocarbon Disk Interposition [file sj-pdf-1-han-10.1177_15589447211040879.pdf]

Supplemental material

Linear multivariable regression models for different strength measurements.
For all modesl, the FCR technique is baseline.

Linear multivariable regression models for KEYPINCH

Coefficients[a]

Model		Unstandardized Coefficients		Standardized Coefficients	t	Sig.	95.0% Confidence Interval for B
		B	Std. Error	Beta			Lower Bound
1	(Constant)	8.556	1.807		4.736	.000	4.962
	Treatment code	-1.276	.418	-.298	-3.056	.003	-2.107
	Male/Female	1.975	.391	.476	5.055	.000	1.198
	"bilateral operated	.134	.385	.033	.348	.729	-.632
	Age at operation (in years)	-.074	.021	-.331	-3.554	.001	-.116
	Follow up (in years)	.030	.150	.019	.199	.843	-.269
	Dominant hand operated	-.203	.373	-.050	-.544	.588	-.944

Linear multivariable regression model for TIPPINCH

Coefficients[a]

Model		Unstandardized Coefficients		Standardized Coefficients	t	Sig.	95.0% Confidence Interval for B
		B	Std. Error	Beta			Lower Bound
1	(Constant)	7.964	1.325		6.013	.000	5.329
	Treatment code	1.225	.306	.329	4.001	.000	.616
	Male/Female	1.778	.286	.492	6.206	.000	1.208

"bilateral operated	-.104	.282	-.029	-.367	.715	-.665
Age at operation (in years)	-.080	.015	-.413	-5.253	.000	-.111
Follow up (in years)	-.056	.110	-.042	-.511	.611	-.276
Dominant hand operated	.276	.273	.078	1.010	.316	-.268

Linear regression model for GRIP strength

Coefficients[a]

Model		Unstandardized Coefficients		Standardized Coefficients	t	Sig.	95.0% Confidence Interval for B
		B	Std. Error	Beta			Lower Bound
1	(Constant)	52.938	10.475		5.054	.000	32.097
	Treatment code	5.136	2.421	.179	2.121	.037	.318
	Male/Female	17.227	2.265	.619	7.605	.000	12.719
	"bilateral operated	-1.298	2.231	-.047	-.582	.562	-5.738
	Age at operation (in years)	-.472	.121	-.315	-3.906	.000	-.713
	Follow up (in years)	-.762	.872	-.074	-.874	.385	-2.497
	Dominant hand operated	1.958	2.161	.072	.906	.367	-2.340
